# Brachy-ing Unresectable Endometrial Cancers with Magnetic Resonance Guidance

**DOI:** 10.7759/cureus.2274

**Published:** 2018-03-05

**Authors:** Jessica L Conway, Jelena Lukovic, Stephane Laframboise, Sarah E Ferguson, Kathy Han

**Affiliations:** 1 Radiation Oncology, University of Toronto; 2 Obstetrics and Gynecology, University of Toronto

**Keywords:** endometrial cancer, magnetic resonance imaging, brachytherapy, adaptive radiotherapy, unresectable endometrial cancer, image guided brachytherapy, interstitial brachytherapy

## Abstract

The standard treatment for endometrial cancer is upfront surgery. However, surgical resection is challenging in locally advanced cases extending to the vagina, bladder, bowel, rectum, or parametria. Historically, these cases were managed palliatively due to the inability to escalate radiation dose safely and accurately, and there is a paucity of literature supporting definitive radiation for these patients. Technological advances in brachytherapy, including magnetic resonance guidance (MR-guidance) and interstitial techniques, have improved target precision and dose escalation without additional toxicity to permit curative management. We report two cases of unresectable, locally advanced endometrial cancers treated with MR-guided radiation.

## Introduction

Endometrial cancer is the most common gynecological cancer in North America. Most cases are diagnosed at an early stage; 80% of women present with uterine-confined disease and have a favorable prognosis [1]. Surgery is the standard of care and consists of a total hysterectomy with bilateral salpingo-oophorectomy with or without lymph node dissection. Depending on clinical and pathologic risk factors, adjuvant treatments may be indicated. In some patients, up-front surgical resection is precluded due to medical comorbidities and/or disease extension to the vagina, bladder, bowel, rectum, or parametria. Neo-adjuvant or definitive radiation can be used in unresectable endometrial cancers. However, the prognosis is poor with an average survival of two to eight months [1].

Treatment decisions for surgically unresectable patients are often based on data extrapolated from medically inoperable, recurrent, or adjuvant settings. The American Brachytherapy Society (ABS) developed consensus guidelines for medically inoperable cases [2]. Literature on surgically unresectable endometrial cancer is sparse and heterogeneous, making it challenging to apply to modern techniques [3]. Although there are limited data on the outcomes of localized unresectable stage III patients, ten-year crude local failure rates of 65% (32/49) to 67% (4/6) have been reported [4-5].

Compared with 2D techniques, magnetic resonance-guided (MR-guided) 3D brachytherapy in cervical cancer has improved target delineation and local control, and reduced toxicity [6]. Soft-tissue contrast resolution with MRI is superior to computed tomography (CT) and other imaging modalities, enabling better visualization of tumor and associated structures, such as parametria. Combined intracavitary/interstitial techniques further improve the therapeutic ratio over intracavitary brachytherapy alone in cervical cancer, by escalating tumor dose without exceeding organ at risk (OAR) constraints [6]. MR-guidance and interstitial techniques permit definitive management of unresectable endometrial cases, which were previously treated with less precision, lower doses, or palliative intent. However, access issues, technical expertise, and cost have limited the routine uptake of MR-guided brachytherapy within North America, even for cervical cancer with a 34% utilization of MRI in the United States [7].

There is a paucity of information on the management of unresectable endometrial cancers with radiation and subsequent outcomes. At the Princess Margaret Cancer Centre, we treat unresectable endometrial cancers with neoadjuvant external beam radiation (EBRT) and concurrent cisplatin chemotherapy and if not rendered resectable, brachytherapy using MR-guided combined intracavitary/interstitial techniques. Here we present two cases of unresectable stage IIIB endometrial cancers treated initially with EBRT and concurrent weekly cisplatin. In one case, MRI-guided high dose rate (HDR) combined intracavitary/interstitial brachytherapy followed EBRT. In the other case, surgical resection was achieved but a vaginal recurrence later developed and was treated with MRI-guided pulsed-dose rate (PDR) interstitial brachytherapy. Prior to MR-guidance and use of interstitial techniques, the definitive management of these cases would have been suboptimal.

## Case presentation

**Figure 1:**
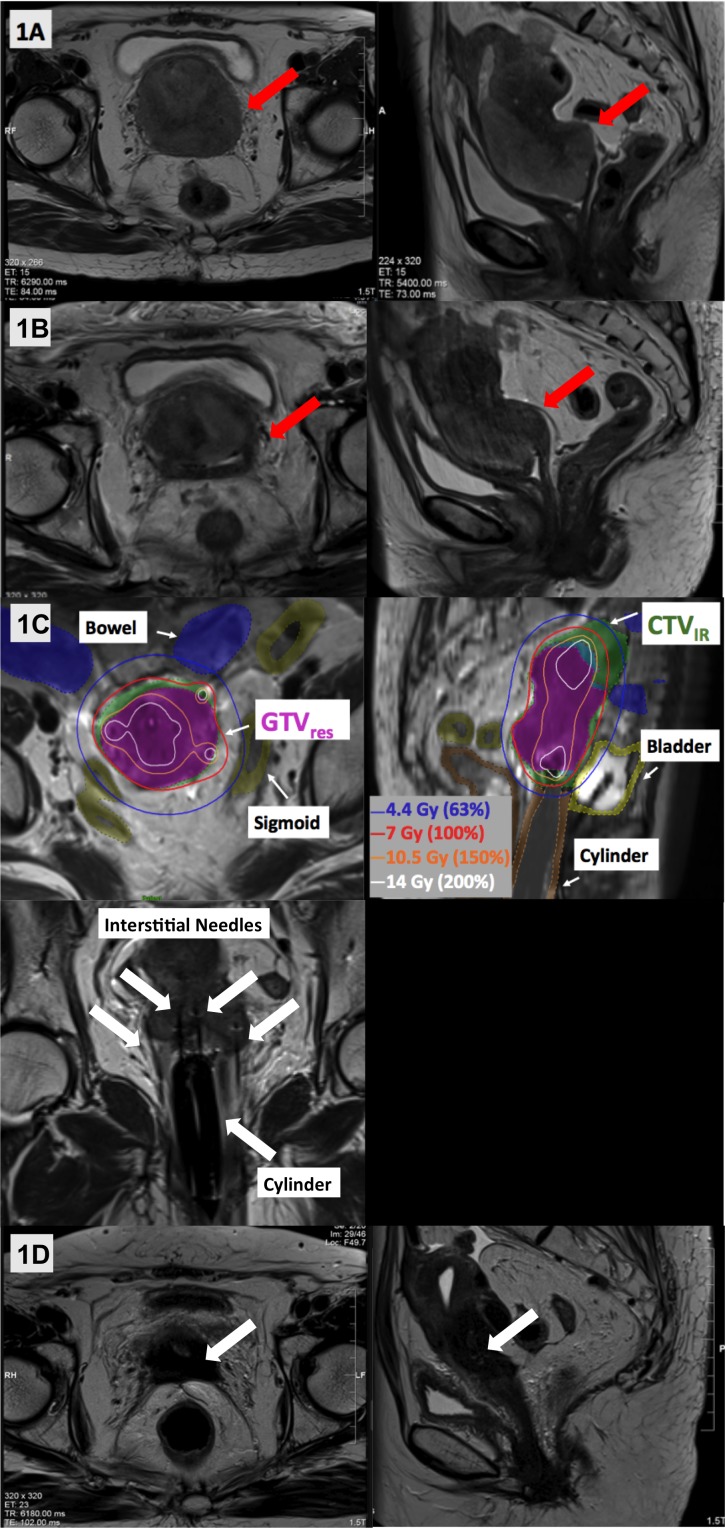
Representative T2-weighted axial and sagittal images acquired across Patient A’s treatment course. (A) Pre-treatment MRI showed a large uterine mass involving the cervix, vagina, and left parametria (red arrows). (B) MRI following external beam radiotherapy showed a good response with reduction in the size of the mass, but persistent parametrial involvement rendering it still unresectable (red arrows). (C) MR-guided brachytherapy was delivered using a template, intra-vaginal cylinder and 12 interstitial needles, with good dosimetric coverage of the residual GTV (GTVres: solid magenta), high-risk CTV (CTVHR: overlaps with GTV res) and intermediate risk CTV (CTVIR: solid green). (D) MRI at 14 months post-brachytherapy showed a complete response, with fibrosis and no residual tumor (white arrows). CTV_HR_ = High risk clinical target volume; CTV_IR_ = Intermediate risk clinical target volume; GTV_res _= Residual gross tumor volume at brachytherapy

Patient A is a 73-year-old, post-menopausal, previously healthy female who presented with a surgically unresectable, clinical stage IIIB endometrial cancer. On physical examination, there was an exophytic cervical mass extending down the upper vagina, and involving the left parametria. Staging MRI showed a 7.4 x 6.4 x 6.9 cm mass extending down the uterus to involve the entire cervix, full-thickness myometrium and left parametria (Figure 1A). Staging CT scans showed no extra-pelvic disease. Endometrial and cervical biopsies confirmed grade 2 endometrioid adenocarcinoma; immunohistochemistry markers were equivocal but radiographically endometrial origin was favored. EBRT (45 Gy in 25 fractions) was delivered to the pelvis with weekly concurrent cisplatin. Clinical examination and MRI at the end of EBRT confirmed persistent left parametrial disease, which remained unresectable (Figure 1B); and therefore, brachytherapy was planned.

A pre-plan for implantation was generated using MRI images acquired with an intra-vaginal cylinder. Following that, a central tandem, intra-vaginal cylinder and Syed-Neblett template (Best Medical, Springfield, USA) with 12 interstitial needles were implanted twice (a week apart). HDR brachytherapy of 28 Gy in 4 fractions (2 fractions per implantation) was delivered. High-risk clinical target volume (CTV_HR_), intermediate risk CTV (CTV_IR_) and OAR volumes were contoured on T2-weighted MRI by adapting the principles of GEC-ESTRO recommendations on 3D image-based treatment planning in cervix cancer brachytherapy [8]. For each brachytherapy fraction, an MRI scan was repeated with the applicator in situ to enable needle adjustment and adaptive planning, with improved visualization of the residual tumor and OAR geometry. Plans were generated in Oncentra (Elekta, Stockholm, Sweden) and evaluated using ABS planning goals [2]. Cumulative dosimetry and planning aims are shown in Table 1 and Figure 1C. If only historical, conventional 2D techniques or CT were available, visualization of residual disease and brachytherapy treatment would have been suboptimal with lower doses to the tumor and less sparing of OARs. In this case, follow-up MRIs up to 14 months showed no residual disease (Figure ID), although PET/CT imaging at 17 months showed two FDG-avid peritoneal nodules outside of the radiation treatment volume. The patient had no severe toxicity when last assessed at 18 months post-treatment.

**Table 1:** Dosimetry and planning aims for Patient A and Patient B *The OAR doses include combined doses from prior EBRT and vaginal vault brachytherapy, and the interstitial brachytherapy. The target doses include only the interstitial brachytherapy component delivered at the time of recurrence. ABS = American Brachytherapy Society; BT = Brachytherapy; CTV_HR_ = High Risk Clinical Target Volume; CTV_IR_ = Intermediate Risk Clinical Target Volume; D2 _cm3_ = Minimum dose delivered to the most exposed 2 cm^3^ of the volume; D_x__% _= Minimum dose delivered to X% of the volume; EBRT = External Beam Radiation; EQD2 = Minimum combined EBRT and brachytherapy biological equivalent 2 Gy doses; GTV_res_ = Residual gross tumor volume at brachytherapy; OAR = Organs at risk

	Patient A EBRT/BT EQD2 Doses	Patient B Total Doses* (interstitial BT dose alone for OAR)	ABS Planning Aims [2]
GTV_res_ D_98 __% _(Gy_10_)	80	79.1	GTV 80 - 90
CTV_HR_ D_90 __%_ (Gy_10_)	89.5	95.7	CTV 70 - 75
CTV_IR_ D_90 __%_ (Gy_10_)	66.4	47.2	
Bladder D2 _cm3_ (Gy_3_)	78.6	55.9 (9.9)	< 90
Rectum D2 _cm3_ (Gy_3_)	61.5	61.5 (15.5)	< 70
Sigmoid D2 _cm3_ (Gy_3_)	70.7	48.5 (2.5)	< 70

**Figure 2:**
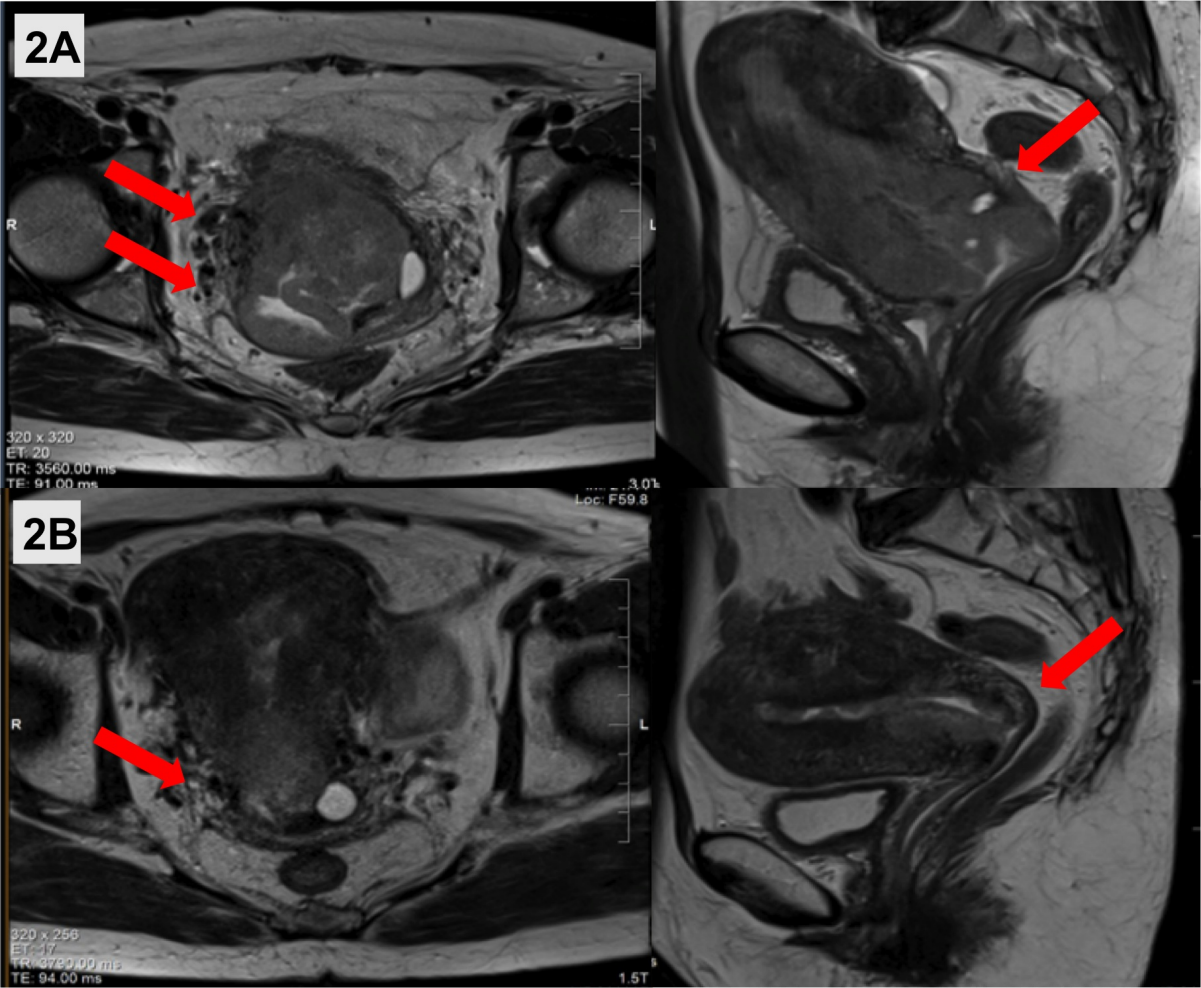
(A) Pre-treatment T2-weighted axial and sagittal MRI showed a large uterine mass extending down the cervix and involving the right parametria (red arrows). (B) MRI at the end of external beam radiotherapy showed a good response with minimal residual parametrial involvement (red arrows), rendering the tumor resectable.

Patient B is a 56-year-old peri-menopausal, previously healthy female who presented with a surgically unresectable, clinical stage IIIB endometrial cancer with a bulky cervix and uterus. A cervical biopsy showed grade 3 endometrioid adenocarcinoma of the endometrium. MRI showed a 5.2 x 5.8 x 7.1 cm uterine mass involving the cervix and right parametria (Figure 2A). Staging investigations revealed no distant disease. She completed EBRT (46 Gy in 23 fractions) with weekly concurrent cisplatin. MRI showed good treatment response with minimal residual right parametrial extension (Figure 2B). A radical abdominal hysterectomy and bilateral salpingo-oophorectomy and infracolic omentectomy were performed one month following EBRT. Final pathology demonstrated partial pathological response, with residual 4.5 cm lower uterine disease involving < 50% myometrium and cervix, and negative parametria (ypT2NX). Since less than 1 cm of vagina was resected, she received adjuvant vaginal vault HDR brachytherapy (11 Gy in 2 fractions) to the upper 3 cm of vagina prescribed to a depth of 5 mm using a 2.5 cm diameter intra-vaginal applicator.

**Figure 3:**
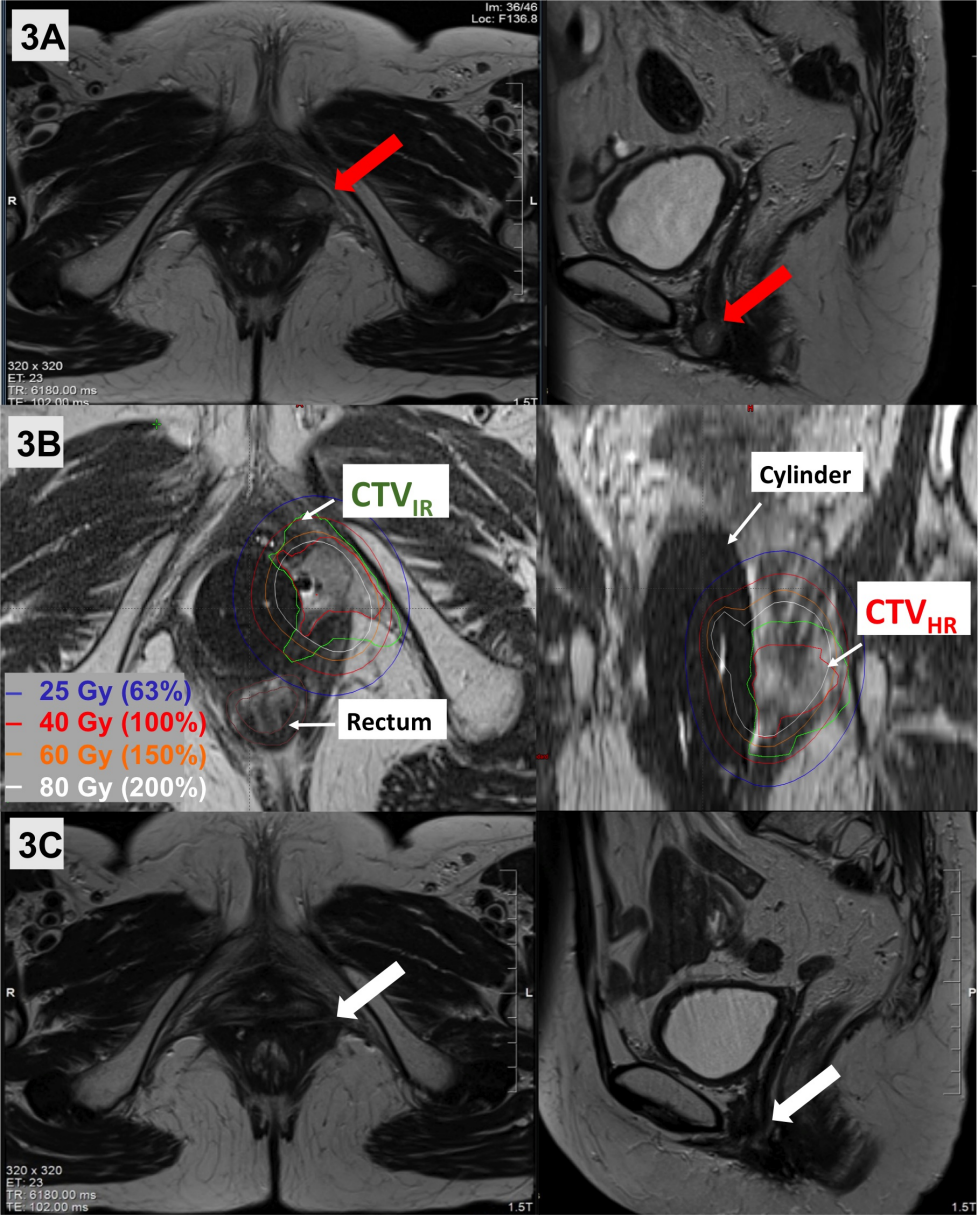
(A) Representative T2-weighted axial and sagittal images of Patient B’s lower vaginal recurrence (red arrows). (B) MR-guided salvage brachytherapy treatment was delivered. using a customized vaginal mold and oblique needles, with excellent coverage of the GTVres (magenta line), CTVHR (overlaps with GTVres) and CTVIR (green line). (C) MRI at 35 months post-brachytherapy showed no residual disease (white arrows). CTV_HR_ = High risk clinical target volume; CTV_IR_ = Intermediate risk clinical target volume; GTV_res _= Residual gross tumor volume at brachytherapy

She developed an isolated lower vaginal recurrence 12 months later measuring 1.5 x 1.2 x 2.1 cm on MRI with restricted diffusion and gadolinium enhancement (Figure 3A). Biopsy confirmed recurrent grade 3, endometrial endometrioid adenocarcinoma. As further surgery would have necessitated an exenteration, a customized vaginal mold was made to guide oblique interstitial needles through the recurrent tumor. Prior to MR-guidance and interstitial techniques, management of this case would have been suboptimal or even palliative (especially given the overlap with previous radiation) and likely associated with increased late toxicity. Here, a brachytherapy plan was simulated using an MRI. An MRI was repeated after insertion of the vaginal mold and interstitial needles for target and OAR delineation and PDR brachytherapy planning (prescription dose of 40Gy) in Brachyvision (Varian, Palo Alto, USA). In this re-treatment setting, MRI was essential to accurately delineate target volumes to avoid overlap from previous treatments; without it, OAR doses would have exceeded constraints due to marginal overlap with previous treatments. Treatment planning was based on the same principles as above and dosimetry took into account previous OAR doses (Table 1 and Figure 3B). MRI scans continue to show stable post-treatment changes at 40 months post-treatment indicating complete radiographic response (Figure 3C). The patient remains without evidence of disease at a follow up 40 months post MRI-guided, salvage interstitial brachytherapy, with mild grade 2 radiation proctitis and grade 1 vaginal toxicity.

## Discussion

Locally advanced, unresectable endometrial cancers are challenging to manage. There is a paucity of published literature and the available data are heterogeneous in terms of treatment intent and technique [3]. Data from cervical cancer are often extrapolated and applied in this setting. Here we present two cases of patients diagnosed with unresectable stage IIIB disease who, without MR-guidance and use of interstitial needles, would have had suboptimal treatment with the compromise of target coverage and/or OARs constraints.

MR-guidance and interstitial brachytherapy techniques facilitate treatment of unresectable endometrial cancer by improving target and OAR delineation as well as the therapeutic ratio [6]. MRI enables confirmation of applicator and interstitial needle placement, better delineation of targets and OAR volumes as well as an assessment of uterine wall thickness/size and cervical or parametrial involvement. Despite this, avoiding OARs and achieving adequate target coverage with large tumor volumes with standard brachytherapy techniques remains a challenge. Interstitial needles facilitate dose escalation as well as OAR avoidance. In our cases, we used 12 and three interstitial needles for patient A and B, respectively. Brachytherapy would not have been achievable without MR-guidance to ensure adequate and safe positioning of interstitial needles.

With larger treatment volumes and dose escalation in unresectable endometrial cancer, it is imperative to evaluate late toxicity. Based on a review on image-guidance for HDR brachytherapy, severe late (> grade 3) complication rates with 3D techniques are 0% compared with up to 21% with 2D planning [5]. There is further promise for MR-guidance to reduce late complications with more accurate OAR delineation. In our case series, Patient A had an asymptomatic sacral insufficiency fracture on MRI at 14 months and Patient B grade 2 proctitis. Neither patient has a severe toxicity, which may reflect the use of image guidance and adaptive brachytherapy.

Data are limited on outcomes for unresectable endometrial cancers managed with radiation with or without a completion hysterectomy. A review article reported local control amongst all stages of unresectable endometrial cancers of 90%–100% with 3D planning in comparison to 70%–90% with 2D planning [3]. However, only two small case series with a combined total of 65 patients used 3D brachytherapy; these series included only three stage III patients and five purely MRI-planned cases; in the latter category, none were Stage III [9-10].

A few, small 2D-era series have reported much lower local control rates in unresectable stage III patients of 33–35% [4-5]. Extrapolating from cervical cancer and the limited publications on HDR brachytherapy in endometrial cancer, there is a suggestion of improved local control and toxicity with 3D planning. In our cases, with the use of both MR-guidance and 3D planning, we report good local control without severe toxicity.

## Conclusions

The combination of EBRT and MR-guided interstitial brachytherapy is a feasible treatment option for women with locally extensive unresectable or recurrent endometrial cancer. We demonstrate favorable local control and limited toxicity in two patients treated with this approach.
